# The impacts of natural polyphenols and exercise alone or together on microRNAs and angiogenic signaling

**DOI:** 10.3389/fphar.2025.1560305

**Published:** 2025-06-24

**Authors:** Yanna Sun, Linlin Chen, Lili Xiao, Xiaofang Wang, Jamal Hallajzadeh

**Affiliations:** ^1^ Department of Cardiology, The First Affiliated of Zhengzhou University, Zhengzhou, Henan, China; ^2^ Research Center for Evidence-Based Health Management, Maragheh University of Medical Sciences, Maragheh, Iran

**Keywords:** polyphenols, angiogenesis, miRNAs, exercise, effect

## Abstract

microRNAs (miRNAs) are a class of small noncoding RNAs that regulate gene expression at the RNA level. In recent decades, increasing evidence has shown that miRNAs play crucial regulatory roles in various biological processes and are considered promising targets for preventing and treating several diseases, including cardiovascular disorders. Multiple studies have suggested that miRNAs serve as significant modulators of angiogenesis. It is believed that the angiogenic response of the vascular endothelium is influenced by miRNAs, indicating a new perspective on the angiogenesis process. Exercise training is an effective strategy for enhancing cardiovascular health, partly due to its positive effects on lipid profiles and increased blood flow in vessels resulting from structural changes in the vasoreactivity of coronary arteries. The literature also provides evidence of polyphenols’ anti-inflammatory, antioxidant, antiviral, and anti-cancer properties across various organs. Polyphenols offer significant health benefits and are recognized for their role in preventing and treating multiple disorders, including cardiovascular disease. They can reduce the risk of ischemic stroke by mitigating platelet aggregation, dyslipidemia, and hypertension. To our knowledge, no current review comprehensively summarizes the combined effects of polyphenols and exercise on angiogenesis. Therefore, in the present review, we examined influence of polyphenols intake and exercise alone or together on angiogenic signaling via modulating the expression of miRNAs.

## 1 Introduction

The interplay between polyphenols and physical exercise has garnered significant attention in health promotion and disease prevention. Both modalities have been shown to benefit a range of biological processes, including oxidative stress, inflammation, and metabolic regulation (Qiu et al., 2023; Paul et al., 2024; Naderi et al., 2019). A particularly intriguing area of research explores the combined effects of miRNAs and angiogenic signaling pathways, essential for maintaining homeostasis and aiding recovery across various physiological contexts.

**FIGURE 1:**
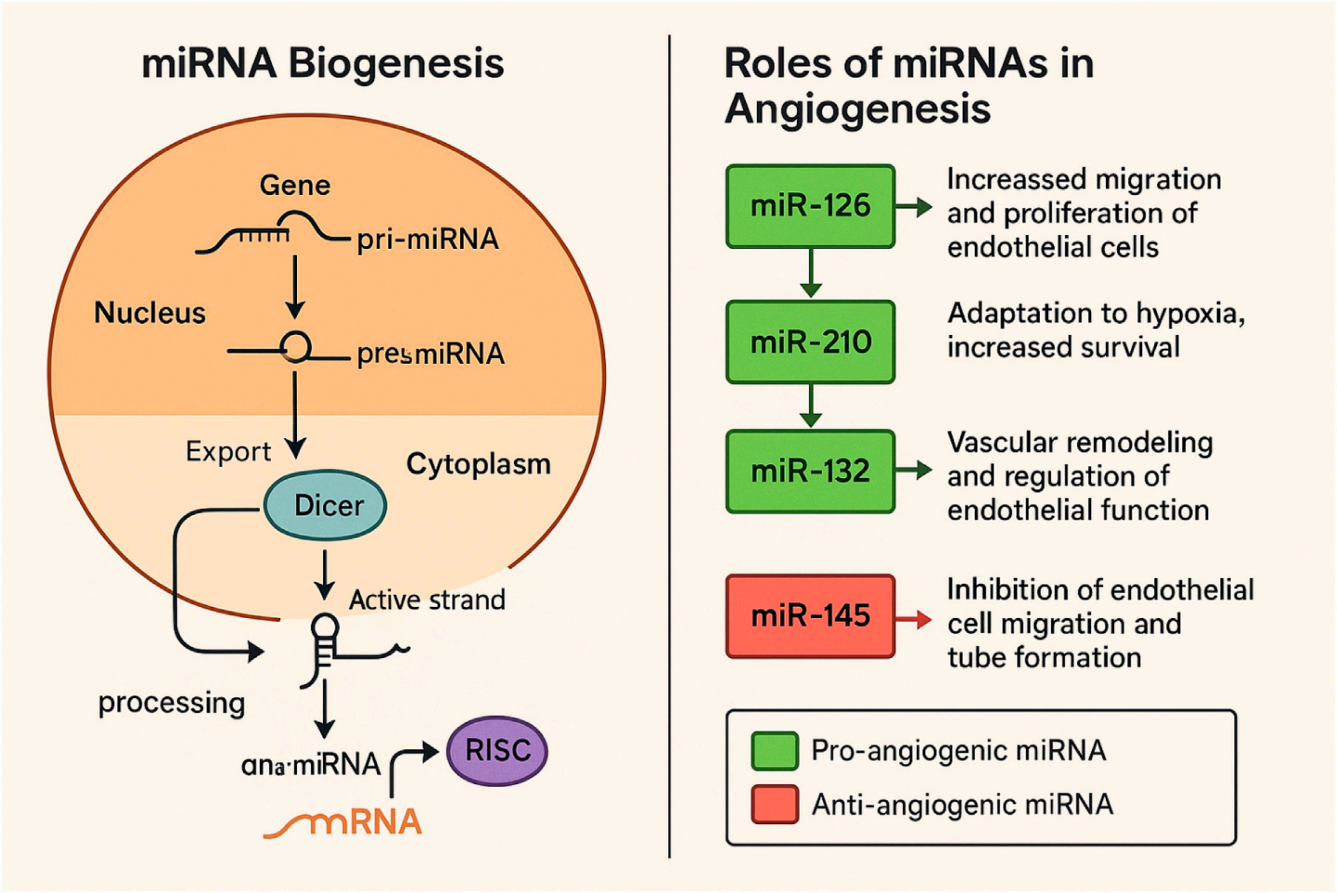
Biogenesis of miRNAs and their diverse roles in angiogenesis.

miRNAs are small, non-coding RNA molecules crucial for gene regulation (Figure 1), impacting cellular functions such as proliferation, apoptosis, and differentiation (Billi et al., 2024). Their capacity to fine-tune gene expression means that dysregulation of miRNAs has been linked to a wide range of diseases, including cancer, diabetes, and cardiovascular disorders (Nemeth et al., 2024; Searles, 2024; Rao et al., 2024). In particular, angiogenesis—the formation of new blood vessels—plays a crucial role in these conditions, as it is vital for tumor growth metastasis and tissue repair after injury (Lorenc et al., 2024; Shi et al., 2023). Both polyphenols and exercise have been shown to modulate miRNA expression, thereby impacting angiogenic signaling pathways and contributing to tissue repair and regeneration.

**FIGURE 2:**
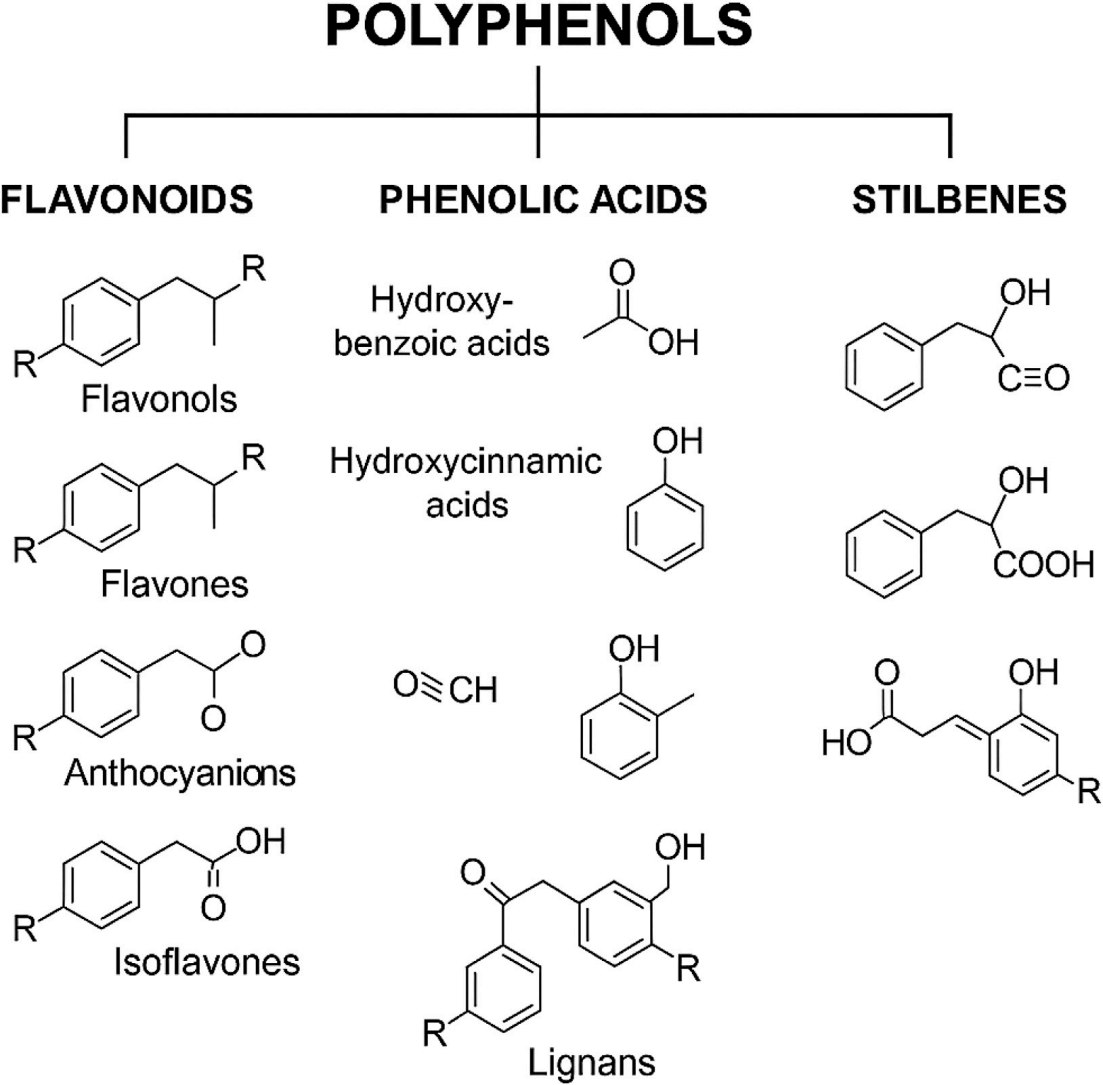
Classification of natural polyphenols.

Polyphenols, naturally occurring compounds found abundantly in plants and fungi (Figure 2), exhibit potent antioxidant and anti-inflammatory properties. Different subclasses of polyphenols, such as flavonoids, phenolic acids, polyphenolic amines, stilbenes, and lignans, target a range of molecular pathways including COX and LOX enzymes, the Nrf2 antioxidant defense system, MAPK signaling, neurotransmitter systems, and estrogen receptor-mediated pathways (Zagoskina et al., 2023; Ciupei et al., 2024; Jomova et al., 2025; Behl et al., 2022; Rebas et al., 2020; Al-Khayri et al., 2023; Singh et al., 2024). Exercise similarly serves as a potent physiological stimulus, enhancing muscle function, vascular remodeling, and systemic resilience (Kan et al., 2018; Hughes, Ellefsen, and Baar, 2018).

**FIGURE 3:**
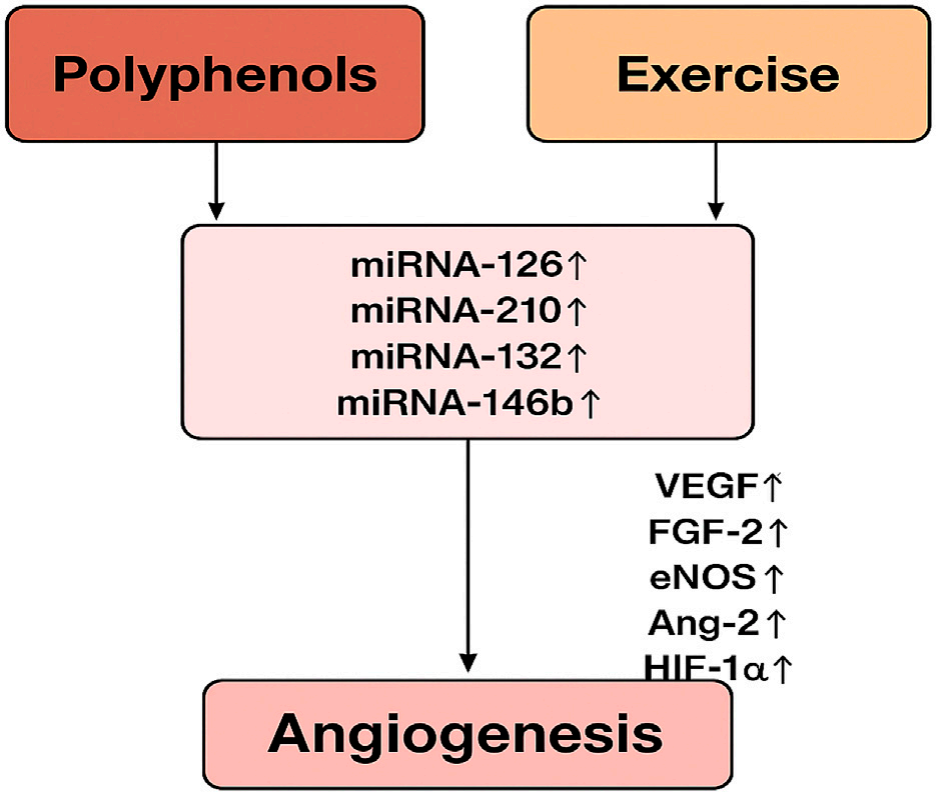
The diagram illustrates the relationship between polyphenols and exercise in regulating key miRNAs involved in angiogenesis. Both polyphenol consumption and exercise are shown to increase the levels of miR-126, miR-210, miR-132, and miR-146b. These miRNAs subsequently enhance the expression of angiogenic factors, including VEGF, FGF-2, eNOS, Ang-2, and HIF-1α, promoting angiogenesis.

It has been shown that exercise and polyphenols alter miRNA profiles, impacting angiogenic processes. Nevertheless, exactly how polyphenols and exercise affect miRNA-mediated angiogenic signaling is still unclear. A coherent diagram that connects these relationships is suggested (see Figure 3), showing how exercise and polyphenol consumption can converge on miRNA control to alter angiogenic results. There is a vast body of research on the effects of polyphenols and exercise separately on angiogenesis and miRNA expression, but less is known about their combined effects. As far as we know, this interaction has not been thoroughly examined in any review. The lack of integrated analysis is shown in Table 1, which provides a chronological summary of important publications examining polyphenols, exercise, miRNAs, and angiogenesis separately. Closing this gap is essential for creating comprehensive cardiovascular health and chronic disease prevention programs. Knowing whether particular miRNAs are involved in the polyphenol-exercise-angiogenesis axis may help identify new biomarkers for therapeutic targeting and health monitoring. By combining knowledge from exercise physiology, nutrition, and molecular biology, future initiatives may promote more proactive and individualized approaches to illness prevention and health promotion. The combination of polyphenol supplementation and exercise training may constitute a paradigm shift in preventive healthcare as studies continue to clarify the complex connections among nutrition, exercise, and molecular signaling networks.

**TABLE 1:** Timeline of key studies (original and review articles) addressing the effects of polyphenols, exercise, or their combination on angiogenesis.

Study	Type	Focus	Combined Mechanistic review?
Ramirez-Sanchez et al. (2012)	Original Research	Epicatechin + Exercise	No
Ringholm et al. (2013)	Original Research	Resveratrol + Exercise	No
Gliemann et al. (2014)	Original Research	Resveratrol + Exercise	No
Mirdar et al. (2014)	Original Research	Curcumin + Exercise	No
Chis et al. (2015)	Original Research	Quercetin + Exercise	No
Ghorbanzadeh et al. (2016)	Original Research	Crocin + Exercise	No
Ghorbanzadeh et al. (2017)	Original Research	Crocin + Exercise	No
Nourshahi et al. (2017)	Original Research	Cinnamon + Exercise	No
Sahafian et al. (2018)	Original Research	Genistein + Exercise	No
Far et al. (2019)	Original Research	berberine + Exercise	No
Banaei et al. (2020)	Original Research	Berberine + Exercise	No
Ghiasi et al. (2020)	Original Research	Garlic + Exercise	No
Dariushnejad et al. (2020)	Original Research	Crocin + Exercise	No
Dehbozorgi et al. (2020)	Original Research	Royal Jelly + Exercise	No
Khosravi et al. (2020)	Original Research	Green tea + Exercise	No
Kouchaki et al. (2020)	Original Research	Curcumin + Exercise	No
Sadeghian et al. (2021)	Original Research	Genistein + Exercise	No
Jalali and Shahidi, (2021)	Original Research	Quercetin + Exercise	No
Abdehvand et al. (2022)	Original Research	Quercetin + Exercise	No
Pouya et al. (2023)	Original Research	Crocetin + Exercise	No
Ghasemi et al. (2023)	Original Research	Resveratrol + Exercise	No
Zangiband et al. (2024)	Original Research	Royal Jelly + Exercise	No
—	Review	Exercise alone and angiogenesis	No
—	Review	Polyphenols alone and angiogenesis	No
—	Review	Combined Polyphenols + Exercise (Mechanistic)	X None found

## 2 Angiogenesis

Angiogenesis is the biological process through which new blood vessels form from existing ones, playing a crucial role in tissue development and repair (Eelen et al., 2020). New blood vessel processes consist of two main stages: vasculogenesis and angiogenesis (Kubis and Levy, 2003). Vasculogenesis refers to the initial formation of blood vessels through the generation of endothelial cells, whereas angiogenesis involves the branching and dividing of these preexisting vessels (Kubis and Levy, 2003). When angiogenesis is unregulated, it can be associated with several disease conditions, including tumors, which often induce excess blood vessel formation to facilitate their growth (Albini et al., 2024). Additionally, chronic degenerative disorders such as arthritis and cardiovascular diseases can arise from abnormal angiogenesis (Dudley and Griffioen, 2023; Caliogna et al., 2024; Zhang, 2024). Moreover, eye diseases like diabetic retinopathy are affected by improper blood vessel growth in the retina (Kour et al., 2024). Multiple factors are essential for regulating angiogenesis.

### 2.1 Growth factors and receptors

Vascular Endothelial Growth Factor A (VEGF-A) is a crucial regulator of angiogenesis (Wiszniak and Schwarz, 2021). VEGF is an essential growth factor that plays a significant role in promoting angiogenesis. VEGF stimulates the proliferation of endothelial cells, helps prevent their programmed cell death, enhances vascular permeability, and promotes cell migration, among other effects. Due to these effects, it actively regulates both normal and abnormal angiogenesis (Melincovici et al., 2018). EGFR (Epidermal Growth Factor Receptor) and IGF-1 (Insulin-Like Growth Factor 1) are essential factors that promote the proliferation of endothelial cells (Sabbah, Hajjo, and Sweidan, 2020; Keller and Schmidt, 2017; Zhang Shiyan et al., 2024). EGFR is a receptor that, when activated by its ligands (such as EGF), triggers a cascade of intracellular signaling pathways, including the MAPK/ERK pathway, which is essential for cell proliferation and survival (Zhou et al., 2015). The activation of EGFR in endothelial cells is vital for the angiogenic process, enabling the body to adapt and respond to various physiological and pathological conditions, such as healing and tumor growth (Lorenc et al., 2024). The signaling pathways activated by IGF-1 binding to IGF-1R are crucial for regulating cell growth, division, and survival, making IGF-1 an essential factor in development and tissue maintenance (Werner, 2023). IGF-1 enhances endothelial cell proliferation and survival, contributing to angiogenesis and vascular repair processes (Zhang X. et al., 2024). CTGF (Connective Tissue Growth Factor) aids in matrix remodeling and the formation of blood vessels (Moon et al., 2020). Mice that lack CTGF expression (either throughout their entire body or specifically in endothelial cells) show significant biological changes (Moon et al., 2020). The absence of CTGF negatively impacts the growth and function of vascular cells, compromising the structure and functionality of blood vessels. The development and formation of blood vessels are negatively impacted, suggesting that CTGF plays a vital role in proper vascular development (Moon et al., 2020). The integrity of the blood-brain barrier or other vascular barriers is compromised, which can seriously affect tissue health and function (Moon et al., 2020).

### 2.2 Transcription factors

HIF-1α is crucial for initiating angiogenesis in response to low oxygen levels, enabling the body to adapt to changes in oxygen availability and maintain tissue viability (Zimna and Kurpisz, 2015). Recent studies have demonstrated that hypoxia and the expression of HIF-1 are crucial in angiogenesis through multiple mechanisms. Firstly, HIF-1 triggers the transcription of various angiogenic genes and their receptors, including PlGF, VEGF, ANGPT2, PDGFB, and ANGPT1 (Greijer et al., 2005). These factors are essential for initiating and maintaining the angiogenic process, as they work together to enhance endothelial cells’ survival, proliferation, and migration. Furthermore, HIF-1 regulates proangiogenic chemokines such as CXCR4 (C-X-C chemokine receptor type 4) and S1PRs (sphingosine-1-phosphate receptors), SDF-1α (stromal cell-derived factor 1α) and S1P (sphingosine-1-phosphate), along with their receptors (Xue et al., 2020). This modulation aids in attracting endothelial progenitor cells to areas experiencing hypoxia, effectively enhancing the local vascular repair and regeneration processes. In addition, HIF-1 encourages the proliferation and division of endothelial cells by affecting genes that control the cell cycle and DNA replication, ensuring an adequate supply of endothelial cells needed to form new blood vessels (Slawski et al., 2024). Beyond these direct effects, HIF-1 is also crucial in orchestrating the remodeling of the extracellular matrix (ECM), which is vital for establishing a supportive environment for forming new blood vessels (Magar et al., 2024). By promoting the production of matrix metalloproteinases (MMPs) and other ECM components, HIF-1 aids in the degradation of existing matrix structures, enabling the migration of endothelial cells into hypoxic tissues. Moreover, HIF-1 coordinates metabolic signals that respond to oxygen availability. This integration ensures that energy production and nutrient availability are aligned with the demands of rapidly proliferating endothelial cells (Magar et al., 2024). The interaction between metabolic adaptation and angiogenic signaling is essential, as it allows cells to survive in low-oxygen conditions while promoting the development of a functional vascular network. In summary, the diverse functions of HIF-1 in angiogenesis emphasize its significance in physiological processes like wound healing and tissue regeneration and highlight its potential as a therapeutic target for pathological conditions, such as cancer and ischemic diseases.

### 2.3 Matrix degradation molecules

MMPs are a family of zinc-dependent endopeptidases that degrade a variety of proteins in the ECM (Wang and Khalil, 2018; Cabral-Pacheco et al., 2020). MMPs are generated by various cell types, including leukocytes, fibroblasts, and vascular smooth muscle (VSM) cells (Wang and Khalil, 2018; Cabral-Pacheco et al., 2020). Their activity is controlled at the mRNA expression level and through an activation process that entails removing the propeptide domain from their inactive zymogen form. MMPs can also affect endothelial cell function and influence VSM cell migration, proliferation, calcium signaling, and contraction. They are involved in remodeling vascular tissue during key biological processes such as angiogenesis (Wang and Khalil, 2018; Cabral-Pacheco et al., 2020).

### 2.4 Maturation factors

Eph receptor tyrosine kinases and their Ephrin ligands constitute an essential signaling system that affects multiple facets of cell function and disease (Darling and Lamb, 2019; Zhu et al., 2024; Pasquale, 2024b). These receptors and ligands are anchored to the cell membrane, resulting in Eph/Ephrin interactions at cell-to-cell contact sites (Pasquale, 2024a). EphB4 and EphrinB2 are essential in vascular development and postnatal angiogenesis (Zhu et al., 2024). Research on their expression and function has associated EphB4/EphrinB2 with processes including endothelial cell growth, assembly, migration, survival, and angiogenesis. The signaling mechanisms involving these molecules are intricate, allowing for bidirectional communication, with signals originating from both the Ephrin ligands and Eph receptors (Salvucci and Tosato, 2012).

## 3 Biogenesis and functions of miRNAs, with an emphasis on their role in angiogenesis

The formation of miRNAs involves several critical steps (see Figure 1). When microRNAs are transcribed from a host gene controlled by their promoters, they are processed intergenically or intragenically from the introns and exons of nuclear DNA. RNA polymerase II or III transcribes segments of specific genes (pri-miRNAs) in lengthy double-stranded clusters known as pre-miRNAs through the primary canonical and/or secondary non-canonical pathways (de Mello et al., 2024). The class 2 ribonuclease III enzyme Drosha and the protein DiGeorge Syndrome Critical Region 8 (DGCR8) connect with pri-miRNAs in the canonical route (Prabhakar et al., 2023). The molecule is broken down into a smaller double-stranded pre-miRNA by this microprocessing complex of DGCR8 and Drosha (Partin et al., 2020). Drosha cleaves the duplex pri-miRNA into a 2 nt3’ overhang on pre-miRNA, whereas DGCR8 identifies regions within the pri-miRNA, such as an N6-methyladenylated GGAC and other short sequence motifs (O'Brien et al., 2018). The protein Exportin five then exports pre-miRNAs from the nucleus to the cytoplasm, where they are released for the Endoribonuclease III Dicer to cleave their stem-loop, producing an even shorter double-stranded molecule (Stavast and Erkeland 2019). The Argonaut protein 2 (AGO2) is loaded with the 5p and 3p strands from the pre-miRNAs 5′and 3′ends, respectively (de Mello et al., 2024). A single strand is then released from the AGO2 after the double strand is unwound. The dynamic environment and function of the cell play a crucial role in determining which strand will stay connected to the AGO2, adding a layer of complexity to the process. But typically, the strand that stays loaded into AGO2 (the guide strand) is the one with lesser 5′stability or 5′uracil. Conversely, AGO2 cleaves the passenger strand (unloaded strand), which is often the 3p one, and cellular processes then destroy it. But typically, the strand that stays loaded into AGO 2 (the guide strand) is the one with lesser 5′stability or 5′uracil. Pre-miRNAs created by Drosha and DGCR8 are also the source of microRNAs produced by non-canonical pathways. Pre-miRNAs created by Drosha, DGCR8, and Dicer activities also produce microRNAs produced by non-canonical pathways (Havens et al., 2012). Nevertheless, Exportin one exports the pre-miRNAs straight to the cytoplasm without Drosha cleavage (Martinez et al., 2017). Alternatively, in this route, the 3p strand serves as the guide strand since the 5p strand is typically not the central strand loaded into the AGO2 due to a 7-methylguanosine (m7-G) cap in the double-strand pre-miRNA (Martinez et al., 2017). In addition, Drosha generates a few pre-miRNAs from endogenous short hairpin RNA transcripts (Yang et al., 2010). Because of their brief length, the AGO2 completes its maturation in the cytoplasm by attaching to both strands and creating a single strand following the trimming of the 5p (Yang et al., 2010). Many miRNAs, with a half-life of a few days to a few weeks, exhibit remarkable stability (Coenen-Stass et al., 2019). This is primarily due to their robust association with Ago proteins, which act as a shield against exoribonucleases. Even when the Ago–miRNA complex eventually dissociates, the Iruka E3 ubiquitin ligase turns over unloaded Ago (Kobayashi et al., 2019). However, this is not the end of the story. Free miRNAs, once unloaded, are subject to exoribonucleolytic degradation, a process that underlines the urgency of this degradation mechanism. miRNAs are vital in regulating angiogenesis, impacting a range of processes. Pro-angiogenic miRNAs, such as miR-126, enhance endothelial cell migration and proliferation, thereby promoting the formation of new blood vessels (Guo et al., 2025). miR-210 plays a crucial role in responding to hypoxic conditions, supporting cell survival and adaptation, which facilitates angiogenesis (Saadh et al., 2025). Additionally, miR-132 is involved in vascular remodeling and the regulation of endothelial function, contributing to overall vascular health (Arcucci et al., 2021). Conversely, anti-angiogenic miRNAs, like miR-145, inhibit endothelial cell migration and tube formation, serving as negative regulators of angiogenesis (Pan et al., 2021; Yang et al., 2010).

## 4 Different exercise types and its physiological mechanisms on miRNAs

Different types of exercise have distinct physiological impacts on angiogenesis, each contributing to vascular health in unique ways. Aerobic exercise, which includes activities like running, cycling, and swimming, enhances blood flow and oxygen delivery to tissues while stimulating the production of growth factors such as VEGF (Ross et al., 2023; Pinckard et al., 2019; Ghahramani and Majd, 2020). Aerobic exercise also promotes the expression of angiogenic microRNAs, resulting in improved capillary density (Dastah et al., 2020; Lou et al., 2022; Zhao et al., 2023). High-intensity interval Training (HIIT), characterized by alternating short bursts of intense activity with periods of rest, increases levels of angiogenic factors like Fibroblast Growth Factor-2 (FGF-2) and enhances mitochondrial biogenesis and vascular remodeling, leading to significant improvements in endothelial function (Banaei et al., 2020; Torma et al., 2019; Li, Zhao, and Yang, 2025). Resistance training, encompassing weightlifting and bodyweight exercises, increases muscle mass, thereby improving local blood flow and stimulating the release of growth factors that contribute to angiogenesis while upregulating miRNAs associated with vascular health (Verdijk et al., 2016; Alves et al., 2025; Mei et al., 2024). Swimming, a full-body workout often regarded as low-impact, enhances cardiovascular fitness and promotes capillary growth in muscle tissues (Mølmen, Almquist, and Skattebo, 2025; Zhao et al., 2023), while also increasing levels of neurotrophins that support angiogenic processes and potentially reducing oxidative stress to benefit endothelial function (Dehbozorgi et al., 2020; Sadeghian et al., 2021). Overall, engaging in various exercise modalities can optimize cardiovascular health and enhance the body’s ability to adapt to physiological demands, highlighting the importance of a comprehensive approach to fitness.

## 5 Effects of polyphenols and exercise in angiogenic signaling

Polyphenols, natural compounds found in plants, have gained attention for their potential health benefits, including their roles in angiogenesis (Cao, Cao, and Bråkenhielm, 2002). Angiogenesis is essential in numerous physiological and pathological contexts, such as cardiovascular diseases, wound healing, and cancer (La Mendola, Trincavelli, and Martini, 2022; Peña and Martin, 2024; Zhang X. et al., 2024). Exercise-induced angiogenesis can greatly enhance cardiovascular and metabolic health, improving muscle glucose uptake, crucial for diabetes prevention (Ross et al., 2023). Here’s an overview of the pro-angiogenic and anti-angiogenic effects of polyphenols and exercise alone and combined (see Table 2).

**TABLE 2:** Effects of polyphenols and exercise alone or together on angiogenic signaling.

Polyphenols	Dose	Exercise modalities	Exercise training modality	Duration	Sample size	Mechanism	Effect type (combined polyphenols and exercise)	Models	Ref.
Garlic	250 mg/kg/day	Voluntary training	Running wheel	For 6 weeks	32 Male Wistar rats	miRNA-210↑, miRNA-126↑	Synergistic	*In vivo*	Ghiasi et al. (2020)
Crocin	50 mg/kg, 6 days a week, for 8 weeks	Voluntary Exercise	Running wheel	For 8 weeks	35 rats	miRNA-210↑, miRNA-126↑	Synergistic	*In vivo*	Dariushnejad et al. (2020)
Crocin	50 mg/kg	Voluntary Exercise	Running wheel	For 8 weeks	40 Male Wistar rats	ERK1/2↑, Akt↑, miRNA-210↑, miRNA-126↑, CD31↑	Synergistic	*In vivo*	Ghorbanzadeh et al. (2017)
Royal jelly	100 mg/kg	Aerobic Training	Treadmill	5 sessions/week for 8 weeks	30 female OVXD rats	miRNA126↑,miRNA-210↑	Additive/Synergistic	*In vivo*	Zangiband et al. (2024)
Royal jelly	100 mg/kg	Aerobic Training	Swimming	3 times/week/for 8 weeks	25 rats with AD	BDNF↔, NGF↔	Additive	*In vivo*	Dehbozorgi et al. (2020)
Berberine	10 mg/kg	Aerobic Training	Treadmill	5sessions/week for 8 weeks	50 rats	VEGF↑, FGF2↑	Synergistic	*In vivo*	Banaei et al. (2020)
Berberine	2 and 15 mg/kg for 5 weeks	Resistance Training	N/A	N/A	40 male Wistar rats	IGF1↑,PDGF↑,VEGF↑	Synergistic	*In vivo*	Far et al. (2019)
Genistein	1 mg/kg/day	Aerobic Training	Swimming	1 session/day, 3 days a week, for 5weeks	48 Female Wistar rats	miRNA-146b↓, miRNA-132↓, TNF-α↓, IL-1β↓, VEGF↓ NF-κB↓, ERK↓, MMP-2↓		*In vivo*	Sadeghian et al. (2021)
Genistein	100 mg/kg	Aerobic Training	Swimming		49 male Wistar rats	NO↑, VEGF↑	Synergistic	*In vivo*	Sahafian et al. (2018)
Quercetin	110 mg/kg, 3 days per weeks, for 6 weeks	Aerobic exercise	Treadmill	5–20 min/day, 5 days a week, for 8 weeks	24 female BALB/c	VEGF-A↓,TIE-2 ↔	Synergistic/Additive	*In vivo*	Jalali and Shahidi, (2021)
Quercetin	30 mg/kg	Aerobic exercise	Swimming	1 h/day, 5 days/week, 4 weeks	80 healthy Wistar albino male rats	MDA↓, PC↓, SOD↑, CAT↑, NOx↓, iNOS↓	Synergistic	*In vivo*	Chis et al. (2015)
Quercetin	0.25 mg for 8 weeks	Interval training	HIIT	1 session for 90 min	30 rats	NF-κB↑, FGF-2↑	Synergistic	*In vivo*	Abdehvand et al. (2022)
Crocetin	30 mg/kg per day	HIIT Training	HIIT	5 session/week for 8 weeks	45 elderly male mice with Diabetes	FGF-2↑, NO↓	Synergistic	*In vivo*	Pouya et al. (2023)
Resveratrol	250 mg/day for 8 weeks	Exercise training	Intense exercise training	60 min/session, 3 days/week, for 6 weeks	43 healthy men	VEGF↔, TIMP-1↔, VEGFR-2↔	Synergistic	Human	Gliemann et al. (2014)
Resveratrol	4 g/kg food	Aerobic Training	Running wheel	N/A	N/A	PGC-1α, VEGF	Synergistic	*In vivo*	Ringholm et al. (2013)
Resveratrol	20 mg/kg	Aerobic Training	Treadmill	1 h/day, 5 days a week, for 4 weeks	40 old male Wistar rats	VEGF↑, adropin↑, FGF-2↑, NO↑,Angiostatin↓	Synergistic	*In vivo*	Ghasemi et al. (2023)
Green tea extract	3 times/week for 8 weeks	Aerobic Training	Treadmill	5 days/week	90 Wistar rats	MMP-2↓, MMP-9↔, VEGF↔	Antagonist/Additive	*In vivo*	Khosravi et al. (2020)
(−)-Epicatechin	1 mg/kg twice daily	Aerobic Training	Treadmill	5 sessions/week, for 8 weeks	25 old, C57BL/6N male mice	CD31↑,HIF1α↑,VEGF↑,VEGFR-2↑	Synergistic	*In vivo*	Ramirez-Sanchez et al. (2012)
Curcumin	100 mg/kg	Endurance Training	Treadmill	5 sessions/week, for 5 weeks	40 female BALB/c mice	miRNA-126↑, Angiopoietin-1↓	Synergistic	*In vivo*	Kouchaki et al. (2020)
Curcumin	30 mg/kg 3 day/week for 8 weeks	Endurance Training	Treadmill	3 times/week for 8 weeks	60 male Wistar rats	VEGF↔	Additive	*In vivo*	Mirdar et al. (2014)
Cinnamon	200 mg/kg/day	Exhaustive exercise	Treadmill	8 weeks	32 male Wistar rats	VEGF↓, SOL muscle Endostatin↑, EDL muscle Endostatin↓	Synergistic/Additive	*In vivo*	Nourshahi et al. (2017)

### 5.1 Garlic

The contributions of garlic and exercise to promoting angiogenesis are complex and involve various molecular mechanisms, primarily through the modulation of specific miRNAs like miR-210 and miR-126 (Naderi et al., 2019). Regular exercise improves cardiovascular function by increasing blood flow, which can stimulate angiogenesis. This is crucial for delivering oxygen and nutrients to tissues, especially in conditions like diabetes. Exercise training has significantly increased the expression of miR-210 and miR-126 in myocardial tissue (Naderi et al., 2019). miR-126 is crucial for endothelial cell function and vascular integrity (Jansen et al., 2013; Chistiakov, Orekhov, and Bobryshev, 2016). miR-126 facilitates angiogenesis by boosting the signaling pathways associated with VEGF (Gao et al., 2021). Under hypoxic conditions, miR-210 is upregulated, facilitating angiogenic responses that improve microcirculation and tissue oxygenation. This is particularly important in situations where tissue perfusion is compromised. The research conducted by Ghiasi et al. demonstrated that voluntary exercise, when paired with garlic supplementation at 250 mg/kg per day, significantly improved myocardial angiogenesis (Ghiasi et al., 2020). This was demonstrated by increased levels of CD31, a marker for endothelial cells, which indicates improved blood vessel formation (Kim et al., 2022). Garlic is rich in bioactive compounds, notably allicin, which possesses antioxidant and anti-inflammatory properties (Shang et al., 2019). These effects contribute to cardiovascular health and can enhance angiogenic processes (Naderi et al., 2019). Garlic consumption has been linked to increased expressions of both miR-126 and miR-210 (Ghiasi et al., 2020; Naderi et al., 2019). Garlic enhances VEGF signaling, which is crucial for angiogenesis (Ghiasi et al., 2020). By improving this signaling pathway, garlic can stimulate the formation of new blood vessels, especially in situations of reduced perfusion. Garlic may act as a hydrogen sulfide donor, a molecule recognized for regulating angiogenesis. Hydrogen sulfide can upregulate miR-126 (Ghiasi et al., 2020), thus further promoting vascular health. The pairing of garlic and exercise produces an additive effect that boosts miRNA expression and encourages angiogenesis (Naderi et al., 2019; Ghiasi et al., 2020). This synergism suggests that utilizing both interventions can improve vascular function and structure beyond the benefits of each alone. Both garlic and exercise significantly promote angiogenesis by modulating critical miRNAs like miR-126 and miR-210 (Ghiasi et al., 2020). Enhancing endothelial function, improving VEGF signaling, and inducing angiogenic responses contribute to better cardiovascular health. Their combined effects enhance these benefits, positioning them as promising strategies for improving vascular health, particularly those at risk for cardiovascular diseases. Additional research is necessary to fully elucidate the underlying mechanisms and optimize these interventions for therapeutic applications.

### 5.2 Crocin

Regular voluntary exercise is linked to beneficial changes in cardiovascular health, including improved angiogenesis (Pinckard et al., 2019). Exercise enhances the expression of VEGF, a key pro-angiogenic factor (Pinckard et al., 2019). Exercise helps lower oxidative stress, which can harm cardiovascular health (Pinckard et al., 2019). This reduction may facilitate better angiogenic responses. Exercise has been shown to upregulate miR-210, which promotes angiogenesis by enhancing cell migration and capillary formation (Dariushnejad et al., 2020). Crocin has antioxidant, anti-inflammatory, and cardioprotective properties (Bastani et al., 2022; Demir et al., 2022). Crocin enhances the expression of VEGF, which is crucial for angiogenesis (Saharkhiz et al., 2021). Crocin administration significantly increases the expression levels of miR-126 and miR-210, which are essential for promoting angiogenesis (Ghorbanzadeh et al., 2017). These microRNAs enhance the signaling pathways involving Akt and ERK1/2, leading to improved angiogenic responses (Ghorbanzadeh et al., 2017). Voluntary exercise stimulates angiogenesis by increasing the capillary network in heart tissue (Ghorbanzadeh et al., 2016). This process is also VEGF-dependent. Exercise further elevates the expression of miR-126 and miR-210, amplifying their angiogenic effects (Dastah et al., 2020; Dariushnejad et al., 2020). The combination of exercise and crocin at 50 mg/kg, 6 days a week for 8 weeks, produces a synergistic effect that significantly boosts angiogenesis compared to either treatment on its own (Dariushnejad et al., 2020). The combination of crocin at 50 mg/kg and voluntary exercise results in a significant increase in Akt and ERK1/2 protein levels, further enhancing angiogenic signaling (Ghorbanzadeh et al., 2017). The study concludes that both interventions benefit heart angiogenesis by modulating key microRNAs and signaling pathways, suggesting potential therapeutic strategies for cardiovascular health.

### 5.3 Royal jelly

Exercise can be divided into different categories, such as aerobic, strength training, and flexibility exercises, each providing distinct health advantages. Aerobic training significantly elevates levels of VEGF and endothelial nitric oxide synthase (eNOS), which are critical for angiogenesis (Pinckard et al., 2019). Exercise enhances the expression miR-210, which is known to promote angiogenesis under hypoxic conditions (Bei et al., 2024), and miR-126, which positively influences the PI3K/VEGF pathway (Fernandes et al., 2012; Gomes et al., 2017). Regular aerobic exercise leads to better lipid profiles and reduces oxidative stress, further supporting vascular health and angiogenesis (Roque et al., 2013). Royal jelly contains antioxidants that help reduce oxidative damage, crucial for maintaining healthy endothelial function and promoting angiogenesis (Oršolić and Jazvinšćak Jembrek, 2024). Consumption of 100 mg/kg of royal jelly elevates levels of VEGF and eNOS, comparable to the effects of exercise (Zangiband et al., 2024). This is linked to its ability to enhance miR-210 and miR-126 levels (Zangiband et al., 2024). Although both interventions offer individual benefits, the combined effect of royal jelly and aerobic training is less significant for specific markers (such as VEGF and eNOS) than their impact when administered separately (Dariushnejad et al., 2020).

Exercise, specifically swimming training, is linked to increased levels of neurotrophins such as brain-derived neurotrophic factor (BDNF) and nerve growth factor (NGF) (Zhang Shiyan et al., 2024; Dehbozorgi et al., 2020). These factors are crucial for neuronal health and can promote angiogenesis by enhancing blood flow and nutrient delivery to brain tissues (Blais et al., 2013). Regular physical activity improves cerebral blood flow, essential for maintaining healthy brain function and supporting the growth of new blood vessels (Yen et al., 2023). Exercise may activate signaling pathways, such as the phosphoinositide 3-kinase (PI3K) pathway (Weeks et al., 2012), which are associated with promoting angiogenesis and neuronal survival. Royal jelly is known for its antioxidant properties, which help reduce oxidative stress in the brain. This stress reduction can lead to improved endothelial function and support angiogenesis. The Dehbozorgi et al. (2020) study found that consuming royal jelly at 100 mg/kg significantly elevated NGF levels in the hippocampus, which is crucial for neuronal growth and survival, indirectly supporting angiogenic processes. Royal jelly has been shown to promote neurogenesis, particularly in the hippocampus, which can enhance the brain’s ability to repair and regenerate (Momeni et al., 2025), further supporting angiogenic signaling. Both exercise and royal jelly consumption play critical roles in strengthening angiogenic signaling through their effects on neurotrophic expression, blood flow improvement, and oxidative stress reduction. While exercise promotes the production of neurotrophic factors and enhances cerebral circulation, royal jelly contributes by increasing NGF levels and providing neuroprotective benefits. However, the study noted that their combined effects did not significantly change BDNF and NGF expression when administered simultaneously.

### 5.4 Berberine

HIIT increases VEGF levels, promoting the proliferation and differentiation of vascular endothelial cells, which is crucial for new blood vessel formation (Banaei et al., 2020). HIIT also elevates fibroblast growth factor 2 (FGF2), which supports endothelial cell proliferation and organization (Banaei et al., 2020). FGF2 plays a crucial role in angiogenesis by activating endothelial cells through its binding to FGF receptors (FGFR1–FGFR4) on their surface (Jia et al., 2021). Regular high-intensity exercise helps decrease levels of caspase-3, a marker of apoptosis (Banaei et al., 2020). This reduction is significant as it mitigates cell death in endothelial cells, thereby enhancing angiogenesis. The exercise protocol improved cardiac function post-reperfusion, as indicated by better echocardiographic indices in the intervention groups than in the control group (Banaei et al., 2020). Berberine supplementation increased the expression of angiogenic factors like VEGF, suggesting its role in promoting angiogenesis in ischemic tissues (Banaei et al., 2020). Berberine has been linked to reduced levels of caspase-3, which contributes to its protective effects on endothelial cells during ischemia-reperfusion injury (Banaei et al., 2020). Combining berberine at 10 mg/kg with high-intensity interval training (HIIT) led to a more significant enhancement in angiogenic signaling than either treatment on its own (Banaei et al., 2020). This synergy enhances the overall angiogenic response, leading to improved recovery of myocardial tissue. HIIT and berberine play crucial roles in enhancing angiogenic signaling by increasing the expression of pro-angiogenic factors and reducing apoptosis. Their combined use maximizes these benefits, suggesting a potential therapeutic strategy for conditions like ischemic heart disease.

Resistance training significantly increased the expression of angiogenic factors such as IGF-1, Platelet-Derived Growth Factor (PDGF), and VEGF (Far et al., 2019). IGF-1 facilitates the development of new blood vessels. IGF-1 does this by upregulating angiogenic factors such as vascular endothelial growth factor (VEGF) through the activation of the MAPK and PI3K/AKT signaling pathways (Ackermann et al., 2012). PDGF is involved in cell growth, and PDGF plays a role in angiogenesis and tissue repair (Jian et al., 2022). Exercise helps counteract the adverse effects of diazinon toxicity, which can impair angiogenesis and neuronal health (Far et al., 2019). Resistance training facilitates recovery and neuroprotection by increasing the expression of VEGF and other growth factors. Supplementation with berberine at 2 and 15 mg/kg for 5 weeks also increased levels of IGF-1, PDGF, and VEGF, indicating its positive impact on angiogenesis (Far et al., 2019). This is particularly important in the context of diazinon-induced damage. Berberine has neuroprotective properties (Sunhe et al., 2024)and can enhance neuronal survival pathways, which may further support angiogenesis in the hippocampus (Nathan et al., 2024). The study indicates that the combination of berberine supplementation and resistance training maximizes the expression of angiogenic factors compared to each treatment alone, illustrating a synergistic effect that could enhance recovery from toxin exposure (Far et al., 2019). Both resistance training and berberine supplementation contribute positively to angiogenic signaling by increasing key growth factors like IGF-1, PDGF, and VEGF. Together, they help reduce the harmful effects of diazinon and promote recovery in brain tissue, underscoring their potential therapeutic roles in enhancing angiogenesis and neuronal health.

### 5.5 Genistein

Regular swimming exercise has been demonstrated to lower VEGF levels in pathological conditions like retinal neovascularization (Sadeghian et al., 2021). This variation from other studies was likely attributed to differences in the test protocols, animal species, or age (Sahafian et al., 2018). Exercise can help inhibit excessive blood vessel formation in the retina. Exercise influenced the expression of miRNAs, specifically miR-132 and miR-146a, which are involved in cellular processes that regulate angiogenesis and inflammation (Sadeghian et al., 2021). These miRNAs can promote cell survival and inhibit apoptosis, affecting overall retinal health. Physical activity reduces inflammatory markers such as IL-1β and TNF-α, which are known to play a role in angiogenesis (Sadeghian et al., 2021). By mitigating inflammation, exercise supports healthier angiogenic responses. Genistein, a phytoestrogen, mimics estrogen’s protective effects, particularly in the retina (Nebbioso et al., 2012). Genistein has antioxidant and anti-inflammatory properties that help counteract oxidative stress and inflammation, which are detrimental to angiogenesis (Nebbioso et al., 2012). Genistein treatment at 1 mg/kg per day was associated with decreased expression of VEGF and MMP-2, which play critical roles in angiogenesis (Sadeghian et al., 2021). This suggests that genistein helps normalize angiogenic signaling pathways disrupted by diabetes. Combined, exercise and genistein treatment yielded more significant improvements in angiogenic signaling than either intervention alone, indicating a synergistic effect (Sadeghian et al., 2021). This combination effectively reduced retinal neovascularization and restored healthier signaling pathways. Both exercise and genistein play crucial roles in regulating angiogenic signaling in the retina, particularly in diabetes conditions and hormonal changes due to ovariectomy. Their combined effects promote a healthier retinal environment by reducing inflammation, oxidative stress, and abnormal blood vessel formation.

Exercise, especially acute swimming for 90 min, significantly increases the levels of NO and VEGF in cardiac tissue (Sahafian et al., 2018). This suggests that physical activity stimulates angiogenic pathways, promoting the formation of new blood vessels. Exercise-induced shear stress enhances NO production, which plays a critical role in vasodilation and preventing platelet aggregation. Physical activity boosts hormonal growth factors, such as growth hormone (GH), which can activate pathways that increase angiogenic factors like VEGF. When administered at 100 mg/kg, genistein did not significantly increase NO or VEGF levels compared to the control and saline groups when used alone (Sahafian et al., 2018). However, it was part of combinations (with exercise) that showed increased levels. Genistein is known to enhance the activity of nitric oxide synthase (NOS), leading to increased NO production (Sahafian et al., 2018). Genistein may enhance angiogenesis when combined with exercise by potentially improving endothelial function. The study (Sahafian et al., 2018) concludes that while exercise alone is a robust stimulus for angiogenic signaling through increased NO and VEGF, adding genistein may enhance these effects, particularly in exercise. However, more research is needed to fully understand the interactive mechanisms and the potential benefits of genistein supplementation alongside physical activity.

### 5.6 Quercetin

The roles of quercetin and exercise in promoting angiogenesis are significant, particularly in cardiovascular health and cancer management (Papakyriakopoulou et al., 2022; Kwak et al., 2018). Quercetin, a flavonoid known for its antioxidant effects (Anand David et al., 2016), has demonstrated strong anti-angiogenic properties. In studies, quercetin supplementation significantly reduced vascular endothelial growth factor-A (VEGF-A) expression, which is crucial for new blood vessel formation (Lupo et al., 2019; Uttarawichien et al., 2021). For example, the combination of quercetin at 110 mg/kg taken three times a week for 6 weeks along with aerobic exercise resulted in a significant reduction in VEGF-A levels compared to the control group, underscoring its potential to amplify the anti-angiogenic benefits of exercise (Jalali and Shahidi, 2021). Quercetin helps neutralize free radicals and reduce oxidative stress (Xu et al., 2019). By lowering oxidative stress in endothelial cells, quercetin creates a more favorable environment for angiogenesis (Jalali and Shahidi, 2021). This is important because oxidative stress can hinder endothelial function and vascular growth. Quercetin at a dosage of 30 mg/kg enhances endothelial function by boosting the availability of nitric oxide (NO), a key factor for vasodilation and the formation of new blood vessels (Chis et al., 2015). Maintaining NO levels supports angiogenesis and enhances blood flow. Quercetin has been shown to decrease inflammatory markers, such as inducible nitric oxide synthase (iNOS) (Chis et al., 2015). Reduced inflammation benefits angiogenesis, as chronic inflammation can impair blood vessel formation. Quercetin at a dose of 0.25 mg for 8 weeks has also been associated with the promotion of FGF-2, an important factor in angiogenesis (Abdehvand et al., 2022). This is particularly relevant for tissue repair following myocardial infarction (MI), where FGF-2 supports the formation of new blood vessels in damaged myocardial tissue (Farooq et al., 2021; Li et al., 2021). Regular aerobic exercise enhances cardiovascular function and blood flow, crucial for stimulating angiogenesis (Pinckard et al., 2019). The mechanical forces produced during exercise can stimulate the expression of angiogenic factors like VEGF and FGF-2 (Abdehvand et al., 2022). Exercise also improves overall metabolic health, enhances immune function, and reduces systemic inflammation, creating an environment more conducive to angiogenesis. Aerobic exercise has been shown to positively influence vascular health by affecting the expression of various factors, including VEGF-A and TIE-2 (Dariushnejad et al., 2023; Dopheide et al., 2016). Some studies, such as those involving sustained ischemia models, reported that quercetin did not significantly improve exercise performance or blood supply. This raises questions about its efficacy in specific contexts, such as peripheral arterial disease (PAD), where quercetin and exercise may not produce the expected angiogenic benefits (Phie et al., 2021). The integrated approach of combining dietary supplements like quercetin with lifestyle modifications such as exercise is promising for managing cancer progression and improving cardiovascular health (Sadighparvar et al., 2020; Khajehlandi and Bolboli, 2024). However, further research remains needed to explore the mechanisms and optimize these combined strategies for clinical applications. It is crucial to understand the interactions between quercetin and exercise, as it underscores the complexity of the research and the need for a comprehensive understanding of the topic. Quercetin and exercise play crucial roles in promoting angiogenesis through complementary mechanisms. Quercetin’s antioxidant properties, ability to enhance NO availability, and reduction of inflammation work synergistically with the benefits of exercise, which improves blood flow and induces angiogenic factor expression (Sumi et al., 2013; Zaheri et al., 2023). Together, they present a multifaceted strategy for enhancing vascular health, particularly in contexts like myocardial infarction and cancer management (Zaheri et al., 2023). Further research is essential to understand their interactions and develop effective therapeutic protocols fully.

### 5.7 Crocetin

Crocetin, derived from saffron, has antioxidant properties that help reduce tissue oxidative stress and inflammation (Zaheri et al., 2023). This is crucial for maintaining a healthy environment for angiogenesis, especially in diabetic conditions where oxidative damage is prevalent. The Pouya et al. (2023) study demonstrated that Crocetin supplementation at 30 mg/kg per day, when paired with aerobic exercise, significantly enhanced the expression of FGF-2. FGF-2 is a key factor in promoting the proliferation and migration of endothelial cells, which are essential for angiogenesis (Jia et al., 2021). The findings indicated that crocetin also influenced NO gene expression (Pouya et al., 2023). Lower NO levels with increased FGF-2 suggest a complex interaction where crocetin may help balance these factors to optimize angiogenesis. HIIT improves cardiovascular fitness by increasing blood flow and inducing shear stress on blood vessels. This physical stimulus is critical for promoting angiogenesis through various signaling pathways. The aerobic exercise regimen significantly increased FGF-2 expression in both prediabetic and diabetic groups (Pouya et al., 2023). This enhancement reflects the ability of physical activity to stimulate angiogenic mechanisms in the heart, counteracting the adverse effects of diabetes. The combination of HIIT and crocetin resulted in significantly lower insulin and glucose levels in the treated groups (Pouya et al., 2023). Improved metabolic profiles are associated with enhanced vascular health and reduced risk of cardiovascular complications. The Pouya et al. study concludes that crocetin and HIIT function synergistically to enhance angiogenesis in heart tissue affected by prediabetes and diabetes (Pouya et al., 2023). The increase in FGF-2 expression and the modulation of NO levels highlight their combined potential to improve vascular health. Therefore, incorporating periodic aerobic exercise and crocetin supplementation could be beneficial strategies for managing angiogenesis-related issues in elderly individuals with prediabetes and diabetes.

### 5.8 Resveratrol

In the context of angiogenesis, resveratrol and exercise play significant roles, albeit through different mechanisms and outcomes. Exercise training is well-documented for its ability to enhance angiogenesis (Li et al., 2022a), evidenced by increased capillary-to-fiber (C:F) ratios and elevated levels of angiogenic factors like VEGF and VEGF receptor-2 (VEGFR-2) (Gliemann et al., 2014). These factors promote new blood vessel formation and improve oxygen delivery to skeletal muscle. Regular physical activity stimulates the release of angiogenic factors, which is particularly vital for older adults, as physiological adaptability changes make efficient oxygen transport critical for maintaining muscle function and overall health (Song et al., 2023). The effectiveness of exercise in promoting angiogenesis is linked to the presence of peroxisome proliferator-activated receptor-γ coactivator-1α (PGC-1α), which mediates the beneficial effects of exercise on muscle capillarization (Gliemann et al., 2014). Exercise-induced shear stress on endothelial cells also normalizes premature senescence, reinforcing the restorative effects of physical activity on vascular health (Gliemann et al., 2014). Resveratrol is a polyphenol known for its antioxidant effects, which are expected to enhance the angiogenic response to exercise (Gliemann et al., 2014). Resveratrol can mimic some cellular effects of physical activity, such as promoting mitochondrial biogenesis (Gliemann et al., 2014) and improving metabolic health (Sergi et al., 2020). However, studies, such as those by Gliemann et al. (2014), suggest that resveratrol supplementation at 250 mg per day for 8 weeks may not promote angiogenesis as expected. Specifically, it did not increase C: F ratios or elevate VEGF levels in muscle tissue, suggesting that resveratrol might counteract some of the positive effects of exercise on angiogenesis (Gliemann et al., 2014). In recent studies, it has been noted that resveratrol negatively affects VEGF expression in cultured adipocytes (Cullberg et al., 2013). Resveratrol effectively blocks the hypoxia-induced increase in VEGF mRNA expression (Cullberg et al., 2013). Commonly, VEGF is upregulated in response to hypoxia to promote angiogenesis. However, resveratrol’s inhibitory effect on this response in adipose tissue may impact vascularization and metabolic processes within that environment. While resveratrol at 20 mg/kg can increase certain angiogenic factors (like VEGF and adropin) and enhance NO production (Ghasemi et al., 2023)—vital for blood flow and endothelial cell proliferation—it did not improve muscle capillarization (Gliemann et al., 2014). In the study by Ringholm et al. (2013), it was discovered that resveratrol supplementation at 4 g/kg of food, whether given alone or alongside exercise training, did not affect VEGF protein levels or the capillary-to-fiber ratio in aged mice. This finding, which challenges the conventional understanding of resveratrol’s effects, underscores the complexity of its actions and raises crucial questions about its efficacy in promoting angiogenic adaptations in aging muscle tissue. Instead, resveratrol appeared to lower the levels of tissue inhibitors of metalloproteinases (TIMP-1), a key factor in regulating the remodeling of the extracellular matrix, potentially disrupting the processes that exercise promotes angiogenesis (Gliemann et al., 2014). The interaction between resveratrol and exercise is complex. While both benefit vascular health, their combined effects may not be additive. Resveratrol’s potential to undermine the angiogenic benefits of exercise raises essential questions about dietary supplements’ roles in conjunction with physical activity. These findings suggest a need for reevaluating the role of resveratrol and similar supplements in promoting vascular health, particularly in aging populations. Understanding the specific pathways influenced by resveratrol could help optimize exercise programs and nutritional strategies. The need for further research is paramount to elucidate how resveratrol interacts with exercise and to clarify its mechanisms. This knowledge could lead to more effective interventions to improve older adults’ quality of life and functional capacity, enabling them to maintain an active lifestyle.

In conclusion, while exercise is a potent stimulus for enhancing angiogenesis, the role of resveratrol is less straightforward. However, it shows promise in supporting endothelial function, offering a hopeful avenue for future research and potential health benefits. Understanding these interactions will be crucial for developing comprehensive strategies for promoting vascular health and combating the effects of aging.

### 5.9 Green tea

The role of green tea extract (GTE) and exercise in angiogenesis presents an interesting dynamic, particularly in cancer and cardiovascular health (Alam et al., 2022; Guo et al., 2023). Epigallocatechin-3-gallate (EGCG), a principal constituent of GTE, is noted for its potential chemopreventive properties (Datta et al., 2022). It can inhibit cell growth and reduce tumor-related factors, particularly vascular endothelial growth factor (VEGF), which is crucial for angiogenesis (Liao et al., 2020). GTE also has been shown to suppress MMPs, specifically MMP-2 and MMP-9, which are involved in tumor invasion and angiogenesis (Tanabe et al., 2023). However, the study by Khosravi et al. (2020) found no significant changes in MMP or VEGF levels following GTE consumption three times a week for 8 weeks, indicating that the anticipated anti-angiogenic effects were not achieved under the experimental conditions.

Several factors could explain the discrepancies between the study by Khosravi et al. (2020) and other studies that report the anti-angiogenic effects of GTE. Variations in the dosage and concentration of GTE can significantly affect outcomes, as different studies may employ varying amounts, leading to various levels of bioactive compounds. Additionally, differences in experimental design—such as the duration of the study, the model organism, or the method of administration (e.g., oral vs intravenous)—can influence results. The bioavailability of GTE components also varies among individuals and experimental models, with factors like food intake, gut microbiota, and genetic differences playing a role. Furthermore, the timing of biomarker measurement after GTE consumption may impact results, as some effects could be transient and not captured if assessed at the wrong time. Variability in GTE formulations, with differing concentrations of active ingredients, may lead to inconsistent results. Lastly, biological differences, including hormonal levels, immune responses, and overall health, can result in different reactions to GTE.

Moderate-intensity aerobic exercise appears to have a dual role in angiogenesis. While exercise may promote angiogenesis in healthy tissues (Song et al., 2023), offering a ray of hope in the fight against cancer. The study noted that MMP-2 levels in cancerous rats engaged in aerobic training were lower than in their healthy counterparts, suggesting decreased angiogenic markers due to exercise. However, no significant differences in MMP-9 and VEGF levels were observed, indicating that the exercise protocol did not substantially alter angiogenesis in these cancerous scenarios (Khosravi et al., 2020). The authors concluded that GTE intake and low to moderate-intensity aerobic training did not significantly influence angiogenesis and metastasis markers in the studied rats. This underscores the need for further investigation into how dietary components and exercise can modulate tumor vascularization and metastasis, but also maintains the hope that exercise can play a significant role in this complex process.

Epicatechin (Epi) has been shown to stimulate myocardial angiogenesis by approximately 30% over control levels when administered at 1 mg/kg twice daily for 15 days (Ramirez-Sanchez et al., 2012). This effect is primarily mediated by activating the eNOS pathway, essential for NO production and a key mediator in angiogenesis. Epi enhances protein levels of vital angiogenic factors such as VEGF and its receptor VEGFR2, which are critical for capillary formation (Ramirez-Sanchez et al., 2012). Like Epi, exercise alone promotes angiogenesis, with effects comparable to those induced by Epi (Ramirez-Sanchez et al., 2012). Epi and exercise activate the VEGF/eNOS/NO signaling pathway, which is integral for new blood vessel formation (Ramirez-Sanchez et al., 2012). Both Epi and exercise activate the VEGF/eNOS/NO signaling pathway, integral for new blood vessel formation (Ramirez-Sanchez et al., 2012). Exercise contributes to increased shear stress on the endothelium, further stimulating angiogenic pathways, while Epi directly activates eNOS, enhancing NO production (Ramirez-Sanchez et al., 2012). Both GTE and exercise have complex roles in angiogenesis. While GTE, particularly through its active compound EGCG, shows potential in inhibiting angiogenesis in certain contexts, its effectiveness may depend on specific conditions. Conversely, Epi and exercise demonstrate a synergistic relationship that enhances myocardial angiogenesis, offering promising therapeutic strategies for ischemic heart conditions. Further research is essential to elucidate these processes’ precise mechanisms and interactions, particularly in cancerous environments and cardiovascular health.

### 5.10 Curcumin

Curcumin, a bioactive compound derived from turmeric (Urošević et al., 2022), and exercise are influential in regulating angiogenesis (Ghorbanzadeh et al., 2022; Li et al., 2022b). This interplay is particularly relevant in cancer treatment and recovery from oxidative stress. Curcumin has been shown to inhibit angiogenesis by modulatingli various signaling pathways (Astinfeshan et al., 2019). In the study by Langroudi and Delfan, (2020), curcumin supplementation at 100 mg/kg significantly decreased the expression of angiopoietin-1, a protein that promotes blood vessel formation.

This reduction suggests that curcumin can effectively regulate angiogenesis, particularly in tumor environments where excessive blood vessel growth is often detrimental. The study highlighted the synergistic effects of combining moderate-intensity exercise with curcumin supplementation (Langroudi and Delfan, 2020). The combination resulted in a significant increase in miR-126 levels and a notable decrease in angiopoietin-1 compared to control groups (Langroudi and Delfan, 2020). This indicates that exercise not only helps manage tumor growth but also enhances the anti-angiogenic effects of curcumin. The findings suggest that the integration of endurance training and curcumin supplementation provides a more pronounced effect on reducing tumor mass and influencing angiogenic factors than either intervention alone. This synergy underscores the potential clinical applications of combining exercise and dietary interventions in therapeutic strategies for breast cancer management, offering a promising integrated approach.

In summary, both curcumin and exercise play significant roles in regulating angiogenesis. Curcumin inhibits pathways that promote blood vessel formation while enhancing the expression of protective miRNAs. Exercise, on the other hand, promotes angiogenesis through increased VEGF levels and improved blood flow. Combined, these interventions synergize, inspiring us to explore new treatment strategies for managing cancer and improving vascular health.

### 5.11 Cinnamon

The research by Nourshahi et al. (2017) sheds light on the intricate roles of cinnamon extract and exercise in the signaling pathways that regulate angiogenesis. Angiogenesis, the process of forming new blood vessels, is vital for tissue recovery and adaptation, particularly in aging populations where vascular health can decline. Cinnamon extract supplementation at 200 mg/kg per day led to a significant decrease in VEGF levels in the soleus (SOL) muscle (Nourshahi et al., 2017). VEGF is a key signaling molecule that stimulates angiogenesis. By reducing VEGF levels, cinnamon extract may inhibit the body’s natural ability to promote new blood vessel formation, which is essential for delivering oxygen and nutrients to tissues, particularly after injury or during recovery (Nourshahi et al., 2017). Concurrently, the increase in Endostatin levels suggests that cinnamon extract enhances the activity of angiogenesis inhibitors (Nourshahi et al., 2017). Endostatin is known to limit excessive blood vessel growth (Walia et al., 2015), indicating that cinnamon may play a dual role by not only lowering a pro-angiogenic factor like VEGF but also increasing an angiogenesis inhibitor (Nourshahi et al., 2017). This inhibitory effect on angiogenesis is particularly concerning for aged rats, as adequate blood supply is crucial for muscle recovery and adaptation (Nourshahi et al., 2017). By potentially restricting angiogenesis, cinnamon extract might impede the healing processes and functional improvements that are necessary for older individuals. In the exhaustive exercise (EX) group, an immediate decrease in VEGF levels was observed following exercise (Nourshahi et al., 2017). This initial drop may reflect the body’s acute response to the heightened oxygen demand and metabolic stress experienced during intense activity (Nourshahi et al., 2017). It suggests a temporary suppression of angiogenesis signaling as the body reallocates resources. Interestingly, VEGF levels increased 4 h post-exercise, indicating a rebound effect that stimulates angiogenesis during the recovery phase (Nourshahi et al., 2017). This response underscores the role of exercise as a potent stimulus for angiogenesis, facilitating the repair of muscle tissue and enhancing blood supply in the aftermath of physical exertion. In the combined cinnamon extract and exercise group, the increase in VEGF levels post-exercise suggests that the angiogenic signaling activated by exercise can prevail over the inhibitory effects of cinnamon extract (Nourshahi et al., 2017). This highlights the dynamic nature of these signaling pathways, where physical activity can activate mechanisms that promote vascular health, even in the presence of compounds that typically inhibit angiogenesis.

## 6 Polyphenols and miRNAs

Polyphenols, including flavonoids, polyphenols, and herbal extracts, can affect the expression of various miRNAs (Tuli et al., 2023). For instance, compounds like curcumin and resveratrol have been found to increase miRNAs that support anti-inflammatory responses while decreasing those related to oxidative stress (Ngum et al., 2023) Many polyphenols possess antioxidant properties, which can help regulate miRNA expression involved in oxidative stress pathways, thereby protecting cells from damage and promoting overall cellular health (Ngum et al., 2023). Certain polyphenols can specifically target miRNAs associated with diseases such as cancer, cardiovascular issues, and metabolic disorders (Ngum et al., 2023; Tuli et al., 2023). For example, polyphenols can restore miRNAs that are downregulated in cancer, potentially helping to inhibit tumor growth.

## 7 Combined effects of polyphenols and exercise on miRNAs expression

Integrating polyphenols and exercise could synergistically affect miRNA expression (Najafabadi et al., 2023; Garelnabi and Mahini, 2014). For example, specific polyphenols might amplify the positive impacts of exercise on miRNAs related to inflammation and oxidative stress (Shargh et al., 2025). Polyphenols could also aid recovery after exercise by influencing miRNAs that govern muscle repair and growth (Campbell et al., 2021). This is especially advantageous for athletes and older individuals aiming to preserve muscle mass and functionality. Polyphenols and exercise offer a holistic method for influencing miRNAs associated with various diseases (see Table 3). This combined approach may strengthen the body’s defense against chronic conditions like obesity, diabetes, and cardiovascular diseases.

**TABLE 3:** Combined effects of polyphenols and exercise on microRNAs.

Polyphenols	Modalities	Sample size	Dose	Exercise protocol	Effect type	Models	miRNAs expression	Ref.
Cinnamon	Exercise training	32 diabetic rats	200 mg/kg	Swimming for 5 session weekly	Synergistic	*In vivo*	miRNA-133a↑, miRNA-21↑	Najafabadi et al. (2023)
Quercetin	Exercise training	40 male LDLr^−/−^ mice on C57BL/6J	100 g/day	Treadmill for 30 min,15 m/m/5 days/week for 30 days	Synergistic/additive	*In vivo*	miRNA-21↑, miRNA-125-b↑ and miRNA-451↓	Garelnabi and Mahini, (2014)
Royal jelly	Exercise training	42 rats	50 mg/kg and 100 mg/kg	Running wheel for 5 weeks (5 days/week)	Synergistic	*In vivo*	miRNA-34a-5p ↓, miRNA-155-3p↓	Lohrasbi et al. (2022)
Genistein	Exercise training	60 female Wistar rats	1 mg/kg/day	Swimming (5–20 min/day) for 5 days	Synergistic	*In vivo*	miRNA-132↑	Habibi et al. (2017)
Genistein	Exercise training	56 rats	1 mg/kg, daily for 8 weeks	Swimming (60 min/day) for 6days/weeks	Synergistic	*In vivo*	miRNA-133↑	Nazari-Serenjeh et al. (2021)
Soy Isoflavone	HIIT	50 female Wistar rats	60 mg/kg/day	HIIT 5 days per week for 6 weeks	Additive	*In vivo*	miRNA-133↑	Mirheidari et al. (2022)

The interplay between curcumin and exercise in influencing miRNA expression is a compelling area of research with significant implications for various diseases, including cardiovascular and inflammatory conditions and cancer. Curcumin has demonstrated protective effects against cardiac injury, particularly in contexts of oxidative stress, such as arsenic exposure (Cox et al., 2022; Lan et al., 2022). Studies have shown that curcumin supplementation can significantly reduce levels of caspase-3, a key enzyme involved in the apoptotic pathway (Majidi et al., 2020; Olanlokun et al., 2022). This reduction indicates a potential mechanism for enhancing cell survival in cardiomyocytes. In experimental setups combining HIIT and curcumin, a significant decrease in miR-1 expression was observed (Majidi et al., 2020). Elevated miR-1 levels are associated with increased cardiomyocyte apoptosis. This suggests curcumin’s ability to downregulate miR-1 may enhance cell survival amid environmental stressors. Concurrently, curcumin increased miR-133 expression and is known for its role in promoting cardiomyocyte health (Majidi et al., 2020). HIIT is known to improve cardiovascular fitness and promote protective mechanisms in cardiac tissues. For example, HIIT has been shown to elevate levels of miR-499 and HSP60 while reducing miR-208, an miRNA linked to increased apoptosis in cardiac cells (Jadidi et al., 2021). The combination of HIIT and curcumin led to decreasing levels of apoptotic markers and enhanced beneficial miRNA profiles, suggesting a robust strategy for improving cardiac health and functional recovery. Curcumin has potent anti-cancer effects, primarily by modulating various signaling pathways involved in cell survival, apoptosis, and proliferation (Cozmin et al., 2024). Notably, it has been shown to reduce miR-21 expression in cancerous tissues (Naghizadeh et al., 2024). miR-21 is recognized as an oncogene, and its overexpression is linked to tumor progression. By downregulating miR-21, curcumin may inhibit tumor growth and promote the expression of tumor suppressor genes, making it a valuable adjunct in cancer therapy. Exercise, particularly endurance training, has also significantly decreased miR-21 levels in cancer models (Naghizadeh et al., 2024). This reduction suggests that physical activity can modulate miRNA profiles and potentially inhibit cancer cell proliferation. The synergy between endurance training and curcumin supplementation in reducing miR-21 levels implies that both strategies may work through complementary mechanisms to enhance anti-tumor effects, thereby improving cancer treatment outcomes (Naghizadeh et al., 2024).

Curcumin is well-documented for its anti-inflammatory properties, particularly in the context of osteoarthritis (OA) (Mende et al., 2025). Curcumin has been shown to significantly reduce inflammatory markers and upregulate miR-130a, which plays a critical role in regulating inflammation and cartilage degradation (Saber et al., 2023). Curcumin may also influence epigenetic changes that affect miRNA expression, suggesting a multifaceted approach to modulating inflammatory responses in OA (Cione et al., 2019). Low-impact exercise, such as swimming, provides mechanical stimulation to joints, enhancing joint function and reducing pain. Similar to curcumin, exercise has been shown to increase miR-130a levels (Saber et al., 2023). The combination of curcumin and exercise leads to a more significant increase in miR-130a levels, which helps restore balance to the disrupted regulatory pathways in OA. Additionally, exercise can reduce HDAC3 expression, further promoting the expression of beneficial miRNAs (Saber et al., 2023). The interplay between curcumin and exercise in modulating miRNA expression offers a promising strategy for addressing various diseases, including cardiovascular issues, cancer, and inflammatory conditions like OA. Their combined effects can lead to favorable shifts in miRNA profiles, promoting cell survival, reducing apoptosis, and enhancing overall health. Future research should continue to explore the underlying mechanisms of these interactions and their clinical applications, potentially leading to integrated therapeutic strategies that leverage both curcumin supplementation and exercise to optimize health outcomes across a spectrum of diseases.

Cinnamon and exercise significantly modulate miRNA expression, particularly related to cardiac health in diabetic conditions (Najafabadi et al., 2023). Cinnamon is recognized for its antioxidant properties, which help neutralize free radicals and reduce oxidative stress, particularly in cardiac tissue (Guo et al., 2024; Padmanabhan and Doss, 2025). This protective effect is linked to the increased expression of miR-133a, pivotal for cardiac protection and inhibiting apoptosis (Najafabadi et al., 2023). The study by Najafabadi et al. (2023) found that cinnamon intake significantly elevated the levels of both miR-133a and miR-21. While miR-133a supports cardiac health, the rise in miR-21—often associated with cardiac damage—suggests a complex role for cinnamon in modulating miRNA profiles. This dual effect indicates that cinnamon may influence both protective and potentially damaging processes in the heart.

Furthermore, cinnamon is believed to activate pathways that enhance mitochondrial biogenesis and reduce inflammation, contributing to a favorable lipid profile and improved cardiac function (Das et al., 2022). These physiological changes are closely tied to alterations in miRNA expression, suggesting that cinnamon supplementation can profoundly impact heart health. Exercise induces physiological adaptations in the heart, including increased oxidative stress, significantly influencing miRNA expression (Najafabadi et al., 2023). The same study indicated that swimming training led to increased expression of miR-133a and miR-21 compared to diabetic control groups (Najafabadi et al., 2023). miR-133a plays a crucial role in cardiac muscle differentiation and protects against apoptosis (Li et al., 2015). Its increased expression in response to exercise indicates a potential enhancement of cardiac resilience, suggesting that physical activity may foster better heart health by promoting the regulation of this critical miRNA (Nie et al., 2016). Although miR-21 is frequently linked to cardiac damage (Nie et al., 2016), its increased expression in exercise may reflect an adaptive response to oxidative stress or a protective mechanism for cardiomyocytes (Zhou et al., 2020). The researchers emphasized the synergistic effects on miR-133a, suggesting that these interventions may collaboratively enhance cardiac health in people with diabetes (Najafabadi et al., 2023). Consistent exercise, paired with cinnamon, can notably affect miRNA expression associated with cardiac function, potentially providing therapeutic advantages for managing diabetes and related cardiovascular risks (Najafabadi et al., 2023). Cinnamon and exercise are crucial in modulating miRNA expression, particularly those linked to cardiac protection. Their combined effects may offer a promising strategy for enhancing cardiac health in diabetic patients.

Quercetin is known for its anti-inflammatory and antioxidant properties, which can significantly impact metabolic processes and gene expression (Aghababaei and Hadidi, 2023). In the study by Garelnabi et al. (Garelnabi and Mahini, 2014), mice supplemented with quercetin exhibited a notable upregulation of miR-21 and miR-125b in both liver and aorta tissues. This indicates that quercetin plays a role in modulating these miRNAs, which are crucial in regulating inflammatory responses and promoting vascular health. Similarly, exercise also has a substantial impact on miRNA expression. The study found that physical activity significantly increased miR-21 and miR-125b levels, with miR-21 showing particularly high expression in response to exercise (Garelnabi and Mahini, 2014). This enhancement suggests that miR-21 could be a biomarker for improved cardiovascular health linked to exercise. Furthermore, the exercise regimen appeared to amplify the protective effects of quercetin on miRNA expression. When quercetin intake was combined with exercise, the highest levels of miR-21 and miR-125b were observed, highlighting a synergistic effect (Garelnabi and Mahini, 2014). This synergy implies that combining these two interventions augments their benefits on miRNA profiles and may enhance cardiovascular protection against atherogenic processes. Interestingly, while both quercetin and exercise were effective at upregulating miR-21 and miR-125b, they were also associated with downregulating miR-451 in the liver (Garelnabi and Mahini, 2014). This suggests a complex interplay in which different miRNAs respond variably to these interventions, pointing to the possibility that miR-451 may have a unique role in the context of atherogenic diets. Overall, the study emphasizes the importance of both quercetin and exercise in modulating miRNA expression, particularly miR-21 and miR-125b, which are relevant to inflammation and cardiovascular health. These findings support the potential therapeutic benefits of integrating dietary interventions with physical activity to enhance metabolic and cardiovascular outcomes.

Royal jelly is rich in bioactive compounds, including 10-hydroxy-2-decenoic acid, which possesses anti-inflammatory and neuroprotective properties (Oršolić and Jazvinšćak Jembrek, 2024). These compounds play a significant role in modulating cellular processes essential for managing symptoms related to multiple sclerosis (MS) (Sabbagh et al., 2025). The study by Lohrasbi et al. (2022) suggested that the intake of royal jelly can influence the expression of specific miRNAs, notably miR-155-3p and miR-34a-5p, which are associated with the pathology of MS. These miRNAs are involved in inflammatory responses and neuroprotection, suggesting that royal jelly may help regulate critical molecular mechanisms associated with MS (Sabbagh et al., 2025). In addition to royal jelly, regular exercise has been shown to improve physical fitness and cognitive function in individuals with MS (Lohrasbi et al., 2022). Exercise can reduce neuroinflammation and foster neuroprotection, which is crucial in combating neurodegenerative diseases (Hu et al., 2024). The interaction between royal jelly and exercise significantly influences the expression of miR-155-3p and miR-34a-5p (Lohrasbi et al., 2022). The research by Lohrasbi et al. (2022) suggested that physical activity can enhance the beneficial effects of royal jelly, leading to improved miRNA profiles that support better management of MS symptoms. The study emphasized that the combination of royal jelly and exercise leads to a more significant modulation of miRNA expression than either treatment alone (Lohrasbi et al., 2022). This synergistic effect is likely to increase the therapeutic potential against the hallmark features of MS, such as inflammation and demyelination. The findings suggest integrating natural supplements like royal jelly with physical activity could represent a valuable non-pharmacological strategy for managing MS. In conclusion, both royal jelly and exercise play essential roles in influencing miRNA expression, which is vital for addressing the underlying mechanisms of MS. Their combined effects may offer a novel therapeutic approach to treating neurodegenerative conditions.

The interaction between genistein, a phytoestrogen found in soy (Rasheed et al., 2022), and exercise presents significant implications for enhancing the expression of various miRNAs crucial for cognitive and cardiovascular health, particularly in postmenopausal women (Yu et al., 2021; Afzal et al., 2024). This discussion focuses on the roles of miRNA-132, miRNA-133, and miRNA-29, examining how these interventions contribute to neuroprotection and cardiac health. Genistein administration in ovariectomized (OVX) rats has been shown to significantly increase the expression miRNA-132, a miRNA essential for neuronal functions such as synaptic plasticity and memory (Habibi et al., 2017). This is particularly important in the context of cognitive decline associated with menopause. The upregulation of miRNA-132 is linked to enhanced levels of BDNF and IGF-1 (Lohrasbi et al., 2022). BDNF and IGF-1 are critical for neuronal health, promoting neurogenesis and synaptic function (Colucci-D'Amato et al., 2020). The activation of BDNF and IGF-1 signaling pathways suggests that genistein may help improve cognitive outcomes in spatial memory tasks, making it a promising candidate for mitigating cognitive decline in menopausal women (Li et al., 2022b). Physical exercise also enhances miRNA-132 expression, BDNF, and IGF-1 levels in the hippocampus (Habibi et al., 2017). Exercise promotes neurogenesis and reduces neuroinflammatory processes that can negatively impact brain health (Liu et al., 2019). The combined effects of genistein and exercise lead to the highest expression levels of miRNA-132, indicating a synergistic relationship that optimizes cognitive functions (Habibi et al., 2017). In diabetic ovariectomized (OD) rats, genistein significantly increased the expression miRNA-133, which is crucial for regulating cardiac hypertrophy and protecting against cardiac dysfunction (Nazari-Serenjeh et al., 2021). miRNA-133 is key in maintaining heart health, particularly in stress conditions such as diabetes (Guo and Nair, 2017; Song et al., 2020). The administration of genistein increased levels of IGF-1 and Bcl-2, promoting cell survival and growth while reducing levels of the pro-apoptotic factor Bax (Nazari-Serenjeh et al., 2021). This shift towards cell survival is vital in preventing excessive apoptosis, which can lead to heart failure.

Additionally, genistein’s antioxidant and anti-inflammatory properties further improve cardiac function. Exercise alone also increases miRNA-133 expression and Bcl-2 and IGF-1 levels (Nazari-Serenjeh et al., 2021). The combination of exercise and genistein amplifies these protective mechanisms, indicating that physical activity can enhance the cardioprotective effects of genistein (Nazari-Serenjeh et al., 2021). The dual intervention upregulates protective miRNAs and reduces oxidative stress and inflammation, critical factors in maintaining heart health. Soy isoflavones, including genistein, have enhanced the expression of miRNA-29 in OVX rats (Mirheidari et al., 2022). miRNA-29 is vital for regulating cardiac function and influences pathways related to fibrosis and apoptosis (Liu et al., 2021). miRNA-29’s role in cellular processes highlights its importance in cardiac health, particularly estrogen deficiency (Ebada et al., 2023). While soy supplementation improved miRNA-29 expression, the results indicated that exercise, particularly HIIT, had a more pronounced effect (Mirheidari et al., 2022). HIIT significantly increased miRNA-29 levels compared to the combination of HIIT and soy. This suggests that while genistein contributes positively, regular physical activity may provide a more direct and robust impact on regulating protective miRNAs involved in cardiac health (Mirheidari et al., 2022). Increased miRNA-29 expression associated with exercise may help mitigate the risks of cardiac diseases in postmenopausal women (Mirheidari et al., 2022). The findings underscore the importance of integrating exercise into lifestyle interventions for improving cardiac health and preventing heart disease. The roles of genistein and exercise in enhancing the expression of miRNA-132, miRNA-133, and miRNA-29 highlight their potential as effective strategies for improving cognitive and cardiovascular health in postmenopausal women. Genistein’s neuroprotective effects, facilitated by increased levels of BDNF and IGF-1 (Singh, Katiyar, and Song, 2025), combined with the beneficial impacts of exercise on neurogenesis and cardiac protection, suggest a multifaceted approach to health management. Together, these interventions optimize the expression of protective miRNAs and mitigate the adverse effects of menopause, emphasizing the importance of combining dietary and lifestyle modifications for optimal health outcomes. This integrated approach may represent a promising strategy for managing at-risk populations’ cognitive decline and cardiovascular health.

## 8 Conclusion

This review emphasizes the significant interplay between natural polyphenols and exercise in modulating miRNA expression and angiogenic signaling. Both interventions independently enhance cardiovascular health and offer synergistic benefits when combined. The evidence suggests that polyphenols, through their antioxidant and anti-inflammatory properties, can amplify the positive effects of exercise on miRNA regulation, particularly in pathways related to angiogenesis. Furthermore, the integration of these lifestyle modifications presents a promising strategy for preventing and managing chronic diseases such as cardiovascular disorders and metabolic conditions. The importance of these findings cannot be overstated, as they provide a deeper understanding of how polyphenols and exercise influence miRNA expression. This knowledge can be used to develop more effective therapeutic approaches that promote overall health and resilience. Fostering a proactive approach that includes dietary interventions and regular physical activity may significantly enhance health outcomes, particularly in populations at risk for cardiovascular and related diseases. This holistic perspective underscores the importance of combining lifestyle modifications with natural compounds to optimize health and wellbeing.

## Glossary

VEGF: Vascular Endothelial Growth Factor
FGF2: Fibroblast Growth Factor 2
CD31: Cluster of Differentiation 31
miRNA: MicroRNA
BDNF: Brain-Derived Neurotrophic Factor
NGF: Nerve Growth Factor
IGF1: Insulin-like Growth Factor 1
PDGF: Platelet-Derived Growth Factor
NO: Nitric Oxide
eNOS: Endothelial Nitric Oxide Synthase
HIIT: High-Intensity Interval Training
NF-κB: Nuclear Factor Kappa B
ERK: Extracellular Signal-Regulated Kinase
TIMP-1: Tissue Inhibitor of Metalloproteinases-1
HIF1α: Hypoxia-Inducible Factor 1-alpha
SOD: Superoxide Dismutase
CAT: Catalase
PGC-1α: Peroxisome Proliferator-Activated Receptor Gamma Coactivator 1-alpha
MMP-2: Matrix Metalloproteinase-2
MMP-9: Matrix Metalloproteinase-9
NOx: Nitric Oxide Derivatives
TIE-2: Tyrosine Kinase with Immunoglobulin-like and EGF-like Domains two
IL-1β: Interleukin-1 beta
TNF-α: Tumor Necrosis Factor-alpha
AD: Alzheimer’s Disease
